# The Injurious Effects of Vegetable and Mineral Acids upon the Enamel of the Human Teeth, with Some Curious Illustrations

**Published:** 1891-08

**Authors:** George W. Weld


### International Dental Journal.

Vol. XII. August, 1891. No. 8.

### Original Communications.[1]

### THE INJURIOUS EFFECTS OF VEGETABLE AND MINERAL ACIDS UPON THE ENAMEL OF THE HUMAN TEETH, WITH SOME CURIOUS ILLUSTRATIONS.[2]

BY GEORGE W. WELD, M.D., D.D.S.

Some one has said that an hypothesis is the life-blood of investigation, and that a person deserves as much credit for destroying an unsound one as he does by establishing a true one. Having had a certain array of questions before me in experimenting upon the effects of acids upon the enamel of the teeth, I shall attempt to put them in such order as may suggest further inquiry in this special direction, as well as to invite your attention to the results of my labor. Every one who has had experience in the chemical laboratory knows that the laws of chemistry are positive.

By the way of simple illustrations, when I pour a small quantity of sulphuric acid ($H_2SO_4$) on a piece of marble (carbonate of lime) we first see an effervescence,—the marble is dissolved, forming the *calcium sulphate* and carbonic-acid gas.

When I pour a small quantity of a six-per-cent. solution of acetic acid into a test-tube, and then add a little of the pulverized

---

[1] The editor and publishers are not responsible for the views of authors of papers published in this department, nor for any claim to novelty, or otherwise, that may be made by them. No papers will be received for this department that have appeared in any other journal published in the country.

[2] Read before the New York Odontological Society, May 19, 1891.

carbonate of lime[1] (common chalk), we will see nearly the same chemical action,—viz., the substance is readily dissolved, forming, in this case, *calcium acetate*, the carbonic-acid gas being freely given off.

But acids act upon different substances with different degrees of energy. Thus it will be observed that when I add a like quantity of the phosphate of lime to the same solution of acetic acid there is no immediate chemical action; the phosphate of lime is held in suspension for a longer period of time. When I use acetic acid of the officinal strength, however, I find that both the carbonate and phosphate of lime are acted upon. If a weak mineral acid be used (a six-per-cent. solution of hydrochloric acid), we will see that both the phosphate and carbonate are immediately dissolved. Here, then, at the very threshold of our investigation, we find a weak vegetable acid which affects easily one of the constituents of the enamel of the teeth,—viz., the carbonate of lime,—while the same quantity of acid of corresponding strength has but little effect on the greater and principal constituent,—viz., the phosphate of lime; and yet both are readily dissolved by acetic acid of officinal strength and by a weak mineral acid. It would seem almost conclusive, from these simple illustrations, when considering the corrosive action of acids upon the enamel of the teeth, that while both the inorganic constituents of the enamel may be injured by weak acids, the first to be affected is that portion that is made up of the carbonate of lime. The effect produced by the same strength of acid on sections of matured enamel, when studied under the microscope, corresponds in many respects with the above experiments. I wish to say, however, at this point, that I shall make no attempt to enter into a study of the finer processes of the development and structure of the enamel. I am willing to leave that to the dental histologist and the skilful microscopist. I am aware that I am dealing with the coarser morphology of the enamel organ, and what I shall say on the subject may or may not affect or determine the presence and supposed existence of a *reticulum* or the former existence of the *enamel cell.* I am tearing down, so to speak, a delicate structure, which is perhaps the most difficult of all structures that the histologist is called upon to investigate. But if, in the process of tearing down and separating the enamel rods by my own method, I shall be fortunate enough to add anything that shall in any way assist

---

[1] The so-called chalk commonly used for black-board purposes is the *sulphate of lime.*

in settling a much mooted question, I shall feel that I have been amply repaid for my trouble. If a section of enamel be placed under a microscope and a small quantity of a twelve-per-cent. solution of acetic acid, by the use of blotting paper, be floated between the glass cover and slide, it will be observed, after a certain length of time, that everything has disappeared excepting a slight *residue*,[1] just as we said, a few minutes ago, the phosphate of lime disappears when using officinal acetic acid. If, however, in the same manner, we use a six-per-cent. instead of a twelve-per-cent. solution, we will find in the course of a few minutes that only one part of the enamel has been effected by the acid,—viz., the cement or interprismatic substance. And we find that we are enabled to separate and isolate the enamel rods without affecting their physical character; but the greater the strength of the acid the wider the spaces between the enamel rods will grow, until at last, if the acid be of sufficient strength, the whole structure succumbs to its influence and is dissolved. If, then, in the test-tube we find that a four-per-cent. solution of acetic acid acts readily on the carbonate but not on the phosphate of lime, while a stronger solution destroys both, and a corresponding and similar action upon the same inorganic matter when under the microscope, it would seem almost conclusive, from a chemical point of view, that the interprismatic substance is composed of the carbonate, and the enamel rods themselves of nothing more nor less than the phosphate of lime. Professor Elliott, who is with us this evening, will, I hope, later on give us the benefit of his experiments on this point.

In marked contrast is the action of weak acids upon the dentine of the teeth. In fact, it may be said that under certain circumstances a weak acid will decalcify the enamel without affecting to any appreciable extent the dentine. Here are a few specimens of the crowns of molar teeth, which were cut transversely across at their cervical portion. It will be observed that the enamel and the dentine have been completely separated, and, although the dentine is hard and apparently unaffected by the acid used, the enamel is soft and can be easily peeled off from the dentine. Drs. Heitzmann and Sudduth are of the opinion, however, that the dentine of these specimens is partially decalcified, although, it must be stated, when they were taken out of the acid solution the dentine was apparently as hard as it is at the present time.

---

[1] This residue is probably silica or the fluoride of calcium unaffected by the acid.

The present appearance of these specimens, the physical condition of the dentine and enamel, and their present relations as dry specimens would seem to indicate that previous to their immersion in the acid there was but little, if any, organic matter existing between the enamel and dentine. At least a magnifying glass of high power fails to reveal at the present time any organic fibres, and the acid[1] employed was very weak, and, I should judge, not of sufficient strength to destroy organic matter had it previously existed.

When I pour a small quantity of strong lactic acid into a test-tube, and add the carbonate of lime, which was dissolved, as we saw, by six-per-cent. acetic acid, we shall observe that the chemical action is extremely slow; but when I add the carbonate of lime to a six-per-cent. solution of the same acid, a solution about eight times less in strength, the carbonate is immediately attacked by the acid and dissolved. The strong lactic acid is thick and viscid, and the particles of the lime carbonate, when immersed in the acid, are doubtless coated over, which prevents chemical action. The same mechanical reason holds good when a tooth is immersed in a solution of acid and simple syrup,—the presence of the sugar in the solution coats the surface of the enamel, which, in the same manner, prevents chemical affinity. It is not always the strongest acid that dissolves the constituents of the enamel; but it is a matter of fluidity and solubility. All the vegetable acids affect the carbonate of lime, and with different degrees of energy the phosphate. Thus, if the phosphate of lime be added to a twelve-per-cent. solution of lactic acid it is very readily dissolved; and the ratio of effects obtained with a twelve-per-cent. solution of three different acids—viz., lactic, malic, and acetic—would seem to show that the lactic acid acts the most powerfully, the malic acid the next, and then the acetic. A six-per-cent. solution of malic acid, however, seems to act somewhat more readily on the carbonate of lime than either the lactic or acetic.

It seems rational to suppose that the enamel of the teeth is affected by acids at the weakest point in the enamel structure, and whether the acid be a mucous acid, or a result of fermentation in the mouth, or introduced from without, the law of affinity is

---

[1] The acid employed was one-half of one per cent. of phosphoric acid, the tooth being allowed to remain in the acid solution for a period of from one to four weeks. But almost any weak mineral acid will probably produce the same results.

always the same.[1] The cervico-labial surfaces of the enamel near the margin of the gums are the thinnest and weakest portion of the enamel, and consequently less capable of resisting the influence of an acid; especially is this the case when the gum has receded, leaving the edge of the enamel wholly or partly exposed. When an acid of any kind is taken into the mouth (supposing the mouth to be thoroughly rinsed with the acid), it will be found that the gums at their marginal portions retain the acid for a longer period of time than either the smooth surfaces of the teeth or the mucous membranes of the mouth. It is the retention of acids by the marginal portions of the gums, in connection with the constant presence of the so-called mucous acid at the same points, which I believe supplements and explains the etiology of erosion. The proof that the margins of the gums retain acids longer than the surfaces of the teeth or the mucous membrane of the mouth is very simple. Let any one first rinse the mouth, and brush the teeth with a strong alkaline solution, being careful to remove any acid mucus that may be present around the margins of the gums; then rinse the mouth thoroughly, first with a weak acid solution, and then water. In the course of a few minutes, or before time enough has elapsed for the formation of any acid mucus at the above-named points, it will be found that neither the surfaces of the enamel, nor any of the mucous membranes of the mouth, show any trace of acid; but apply the blue litmus paper at the margin of the gums and the presence of an acid is detected at once.[2]

By the way of further illustration in this connection, I wish to mention a case that has recently come under my observation and supervision. A patient, whom I had not seen for a period of five years, returned to have his teeth examined. I found, on examination, that his teeth were but little, if any, decayed, and the fillings, both gold and amalgam, appeared to be in good condition, but his front teeth, especially the canine and bicuspids, near their cervico-

---

[1] It may be observed just at this point that when an acid comes in contact with lime-substance, whether in the mouth, in the street, or in the sewer, the destructive effect is the same, and no amount of after washing with an alkali will remedy the loss.

[2] There are only three secretions of the body which are regularly acid in reaction,—viz., the gastric juice, the urine, and the vaginal secretions. The above experiment would seem to indicate that the mucus, always present around the margins of the gums, corresponds in alkaline reaction (in a normal state) with the saliva; and is therefore only acid on account of the margin of the gums retaining the acids which are taken into the mouth from without.

labial portions, were very extensively grooved. In short, it was a typical case of erosion.

Now, this gentleman, about four years ago, was connected in business with the Pennsylvania Railroad Company, and was called upon at one time to supervise the shipping of a car-load of tooth-wash and powder to some western city; and the manufacturer made him a present of some of the wash and powder; and from time to time kept him well supplied. As the wash was very agreeable to the taste and cost nothing, he very naturally used it freely. When he had been informed of the nature of his trouble, he naturally inquired the cause. I told him that the etiology of the trouble was somewhat obscure, but that it was supposed to be due to an acid acting upon the substance of the teeth from without. He then informed me of the wash which he had been using. I asked him to bring me a small quantity of both powder and wash, and I would test them and see if they contained any acid. He did so with the following results: The *powder* was alkaline in reaction, and of course perfectly harmless; but the liquid proved to be acid in reaction, and I believe that the erosion of his teeth was due entirely to the generous use he had made of that wash. I have brought with me a sample of the wash, and will show by the use of litmus paper its acid reaction. It has, as I observed, a pleasant and agreeable taste, being flavored, I should judge, with anise and cloves. I believe that great injury is often done to the teeth by the ingestion of some one of the many proprietary medicines with which the market is so abundantly supplied. It seems rational to believe that the etiology of erosion is, first, the presence of an acid at the cervico-labial surface, that acid being introduced from without and retained at the margin of the gums, and that this acid first attacks the thin and weak surface of the enamel, and then the dentine. In this connection I have already demonstrated that lactic acid, the supposed acid of the mucus of the gums, acts upon the enamel more readily than the other weak acids, excepting, of course, hydrochloric acid.

To revert again to the question of fluidity and solubility, here is a tooth that has been immersed in the strong tincture of the chloride of iron, a mineral acid, for a period of twenty-four hours, and the enamel is not affected in the slightest degree; but another tooth that has been immersed in the tincture largely diluted with water for a period of *ten seconds* shows the surface of the enamel to be materially injured. If a piece of zinc be placed in strong sulphuric acid ($H_2SO_4$), it will be observed that the acid has no effect upon the structure of the zinc, but if a little water be added to the acid the zinc is chemically destroyed. It is the sulphate of zinc, resulting from the first action, insoluble in the concentrated acid, that forms a protecting coat over the surface of the zinc; the addition of water dissolves this protecting sulphate and renders further chemical action possible. In the case of the tooth immersed in the strong tincture of iron a similar action takes place,—viz., the oxide of iron protects the enamel from immediate chemical action, owing to its compact adherence to its surface. To confirm this point, however, that the destructive effect of acids upon the lime salts of the enamel is dependent to a large extent on the fluidity and solubility of a solution, I will illustrate in a manner that may be considered a crucial test. You observed, a few minutes ago, that a weak solution of a vegetable acid dissolved the carbonate of lime very readily. I will now pour into a glass one teaspoonful of the concentrated sulphuric acid, a quantity sufficient to kill any ordinary mortal, and then will add about eight teaspoonfuls of alcohol, and to this solution will add a little of the carbonate of lime; you observe that the carbonate of lime is not disturbed to any very great extent, for there is but little if any chemical action. If, however, I employ one-half the quantity of this acid and the same proportion of water, instead of alcohol, the chemical energy proves very destructive.

The mineral acids are stronger and more destructive to enamel than any of the vegetable acids. Thus, if hydrochloric acid, instead of a vegetable acid, be used in the same proportion with water, we see at once an increased chemical and destructive energy. Hydrochloric acid is the acid contained in the tincture of the chloride of iron, a preparation of iron extensively employed by physicians throughout the civilized world. This form of iron seems to have special virtues which the other preparations of iron do not possess, and, notwithstanding its properties are extremely acid and astringent, it is employed not only in the treatment of anæmia, but also in large doses in the treatment of erysipelas, neuralgia, diphtheria, acute rheumatism, and other disorders which might be mentioned. It acts also as a diuretic; even in ordinary anæmia many authorities prefer it to any other preparation, claiming that its effects are shown more quickly and satisfactorily. These peculiar properties (especially the diuretic effect) are ascribed, by some, to a peculiar ethereal compound resulting from a mixture of the liquor ferri chloridi and the alcohol used in making the tincture, to which compound its odor is due. Almost every authority in materia medica and therapeutics calls attention to the fact that this preparation of iron acts on the enamel of the teeth with destructive energy; also that it is not tolerated by the mucous membrane of weak stomachs. A great majority of physicians and dentists coincide with this view; and not infrequently, for the above reasons, physicians will prescribe other and inferior preparations of iron. If it were expedient or possible for the physician to prescribe alcohol, when administering acids, we would be relieved from further consideration of the subject; but alcohol, or spirits of any kind, for good and sufficient reasons, cannot, as a rule, be used. We are obliged, therefore, to look for some other liquid, as a vehicle, either to diminish the acidity or to entirely counteract this objectionable feature.

I have found that *Vichy water* answers this purpose admirably; favorable mention, however, is made of Apollinaris water. Two reasons exist why the muriate of iron may be administered with Vichy water without injuring the enamel,—first, a chemical reason; second, a mechanical reason. When one or two ounces of Vichy is added to a drachm of the tincture, a slight effervescence takes place, indicating that the alkaline constituents of the Vichy has neutralized the whole or a part of the free acid introduced with the iron. It is to be noted that this preparation of iron always contains an excess of acid,—*i.e.*, more acid than is essential to hold the iron in solution. The acidity of the iron being diminished, of course, there must necessarily be less affinity for the lime salts of the enamel. The use of Vichy water, therefore, in the administration of this preparation of iron simply eliminates an element of destruction,—viz., the free hydrochloric acid,—without impairing in the least degree any of its specific or tonic virtues. But you may naturally inquire, Cannot this acidity be neutralized by other and different alkalies? Most certainly. But the trouble is in using just the right proportions. If too much alkali be used, the iron will be precipitated in the form of the hydrated oxide. I will illustrate by adding a little of the carbonate of lime to a solution of the iron diluted with water.[1] Vichy, on the contrary, seems to possess just enough quantity of the alkalies to dilute and neutralize the free acid without precipitating the iron (unless an excess be used). It is the excesses *in life* which oftentimes result in constitutional troubles. Our blood is always alkaline in reaction; an excess of alkalinity, however, does not tend to increase the red blood corpus-

---

[1] The point of dilution is determined only by mathematics, and reaches into hundred-thousandths of one per cent.

cles, nor improve the general health. The urine, in its normal state, is always acid, but an excess of acid results in a functional trouble. When this form of iron is administered with Vichy, we have simply a preparation of the perchloride of iron, minus the free hydrochloric acid, plus a small quantity of the chloride of sodium, lime, and magnesium.

The mechanical interference is due to the innumerable little bubbles which form and cover the surface of the enamel when Vichy is used as a vehicle. For instance, you will observe that when a tooth is placed in the tincture and water, not a bubble is to be seen upon the surface of the enamel; but if Vichy, instead of water, be used, you will observe that there are thousands of minute bubbles, which completely cover its surface. Now, these minute bubbles on the surface serve as a protection to the lime salts by interfering and preventing contact with the acid solution. This point is beautifully illustrated in the operation of Smee's battery. In this battery silver and zinc are the two metals employed; but the silver becomes coated with hydrogen bubbles to such an extent as to prevent contact with the acid liquid, which in turn interferes with the constancy of its action. Various schemes have been devised to overcome this mechanical interference. For instance, the surface of the silver has been roughened or corrugated in order to facilitate the disengagement of the bubbles of hydrogen. But this form of battery has been discarded, for the reason that in the more recent batteries this mechanical interference of the nascent hydrogen is overcome by the employment of other electrodes. I will leave the subject of polarization, however, to Professor Elliott, who will illustrate the subject on the black-board. The point that I wish to particularly emphasize is that the above-mentioned preparation of iron can be administered by physicians without the possibility of injuring the enamel of the teeth. And the means by which this end is attained, as above described, in no way interferes with the therapeutical effect of this iron compound. On the contrary, I believe the physicians who have prescribed the iron in this manner during the past four years will agree with me when I say that as a restorative agent, by the elimination of the free acid, it is more likely to be assimilated and tolerated by weak stomachs. The addition of a little syrup flavored with winter-green renders the compound more agreeable to the taste.

We will now briefly consider the *indirect action* of acids upon the enamel. By indirect action I mean the possible absorption of acids by the blood, decreasing its alkalinity, and thereby rendering the fluids of the mouth more acid, and, in consequence, a condition of affairs which renders the enamel of the teeth more susceptible to corrosion. An alkali undoubtedly obeys a chemical law and neutralizes free acid, whether in the stomach or in the test-tube. But in this connection it must be borne in mind that the blood is always alkaline in reaction, and while there is reason to believe that its alkalinity may be diminished, there is no authority, so far as I know, that leads me to believe that its character in this respect is ever changed to such an extent as to render the fluids of the mouth abnormally acid. The presence of the alkalies is essential to the oxidation of the organic constituents of this fluid; in fact, as necessary to the life of the corpuscles as water is necessary to the life of a fish, or common air to the life of mankind.

To be sure, the urine in its normal acid state can be rendered alkaline in reaction by the ingestion of large quantities of the alkalies; but when the alkalies are withheld, it returns again to its normal and acid condition. The reason that the urine may be changed from a normal acid to an abnormal alkaline condition is probably due to the fact that the alkalies are chiefly eliminated by the kidneys. But I am unable to find any authority, and I know of no good reason for believing, that the secretions of the salivary glands in their normal alkaline state can be rendered acid by the ingestion and absorption of acids by the blood. And while I am not prepared to say that such a thing is impossible, I believe it to be very improbable. Clinical observation in the future may show that such a thing occurs at certain times under certain physical conditions, but it will then remain for some one to advance the true physiological reasons.

